# Confronting the culture of care: a call to end disrespect, discrimination, and detainment of women and newborns in health facilities everywhere

**DOI:** 10.1186/s12884-020-02894-z

**Published:** 2020-04-28

**Authors:** Emma Sacks, Emily Peca

**Affiliations:** 1 Johns Hopkins School of Public Health, 615 North Wolfe St, E8011, Baltimore, MD 21205 USA; 2 University Research Co., LLC, 5404 Wisconsin Ave, Suite, Chevy Chase, MD 800 USA

**Keywords:** Maternal and newborn health, Respectful maternity care, Quality of care, Health care utilization, Health equity

## Abstract

Quality and respect are increasingly recognized as critical aspects of the provision of health care, and poor quality may be an essential driver of low health care utilization, especially for maternal and neonatal care. Beyond differential access to care, unequal levels of quality exacerbate inequity, and those who need services most, including displaced, migrant, and conflict-affected populations, may be receiving poorer quality care, or may be deterred from seeking care at all.

Examples from around the world show that mothers and their children are often judged and mistreated for presenting to facilities without clean or “modern” clothing, without soap or clean sheets to use in the hospital, or without gifts like sweets or candies for providers. Underfunded facilities may rely on income from those seeking care, but denying and shaming the poor further discriminates against vulnerable women and newborns, by placing additional financial burden on those already marginalized.

The culture of care needs to shift to create welcoming environments for all care-seekers, regardless of socio-economic status. No one should fear mistreatment, denial of services, or detainment due to lack of gifts or payments. There is an urgent need to ensure that health care centers are safe, friendly, respectful, and hospitable spaces for women, their newborns, and their families.

## Comment

Ambitious global goals, such as the Sustainable Development Goals (SDGs), require health to be viewed as holistic and inclusive. Beyond clinical care, attention must be paid to quality, responsiveness, and patient-centeredness. The Lancet Global Health Commission on High Quality Health Systems found that more deaths globally could be attributed to poor quality than access, and care without quality has been called an “empty promise” [1, 2]. Although data on quality still lag far behind data on coverage [2], a growing amount of research has shown that respect and experience of care are equally important aspects of high quality service provision, especially for maternity care [3]. Further, the link has been shown, in many regions of the world, between perceived quality of care and families’ decisions about when and where to seek obstetric and postpartum care [4–7]. A new study from Bayo et al [8], is no exception: perceived poor quality, including the threat of small or in-kind payment for services, was found to be a main driver in the incredibly low institutional delivery rates in one region in South Sudan.

Poor quality care exacerbates inequity: the most vulnerable may be subject to the most disrespectful or inadequate care, which can lead to avoidance or delays in seeking care for those who need it most. Marginalized women and their families, including those who are in geographically isolated and conflict- or disaster-affected areas, may decide that the benefit of receiving sub-standard care is not worth the cost of seeking it. For those who do seek care, many do not reap the full benefit due to lack of full understanding, consent, or participation, including not providing full information to providers for fear of condescension on denial of care [9, 10]. These suboptimal interactions can diminish likelihood of follow-up, adherence to medication or treatment plans, or willingness to return in the future.

Although lack of respectful, dignified care is a worldwide issue [3], those with fewer resources have less recourse. Fewer options and higher costs for where to seek care, inadequate insurance coverage, weak legal systems, gender-biased laws, and lack of knowledge about patient rights all limit women’s ability to access and receive high quality care. Both those needing and providing health care in fragile states and humanitarian emergency settings may be limited in mobility, infrastructure, and security, and may have been subjected to recent violence, loss, and other traumas. Over 60% of maternal deaths and 45% of neonatal deaths worldwide occur in countries with humanitarian crises, and thus quality of care for these women, their families, and providers, cannot be ignored [11–13].

How can improving quality of care and client experiences be used as an ‘accelerant’ instead of a deterrent to care-seeking? While health care workers and policymakers often attribute low facility utilization to “traditions” and “culture,” women themselves often place primacy on the perceived quality of care and will overcome barriers of gender inequity, decision-making power, cultural norms, transport, and geography to access what they perceive as high-quality care [8].

While the level of complexity and need for significant investment may stultify efforts to enact immediate change, starting at the facility door may be a crucial first step. Care-seekers perceive the culture of a health facility, and the expectations on how one must present may be insurmountable for many [8]. Care-seekers are often required to bring items that are lacking in the facility, like soap, clean sheets, or even living blood donors, all of which may even be in addition to payments, or in contexts where health care is supposed to be free at the point of service. Care-seekers may also be expected to bring gifts for staff, such as food, candies, perfumes, or bribes in the form of cash or promises of future transactions. Further, women seeking obstetric or neonatal care may be required to bring clothing and other newborn items, as a demonstration of “worthiness” to receive “modern” clinical care and signify “readiness” to parent. In these quotes, women describe the shaming that occurs at health facilities when expectations around purchasing newborn clothing are not met:

*“However, in the hospital they want us to buy diapers, socks, and blankets so the newborns are all the same… If a woman does not want to buy clothes for her child they say, ‘Well, then you should be operated on so you don’t have any more children since you do not have enough money to take care of your children.’ For this reason I did not want to go to the facility.” –Interview, woman, rural Guatemala* [14]

*“She [the nurse] told me, ‘You didn’t bring clothes for your baby? Do you not care about your child? How can you be a parent if you cannot clothe your baby.’” –Focus group, women, rural Zambia* [15]

For women and families who come to facilities without required items, new evidence suggests they may even be illegally detained, along with those unable to directly pay for services, contributing to the alarming and underreported phenomenon of imprisonment in health facilities [16]:

*“If you deliver in the hospital you have to pay for sweets and soaps which some people cannot afford and so they prefer to deliver from home. If you deliver in the hospital and do not pay for sweets and soaps, you will not be discharged; this needs to be stopped.” –Focus group, women, South Sudan* [8]

Demands for payments may be a way for the facility to raise needed funds for operating costs, and to fill health systems gaps; demands for new clothing may be an opportunity to exploit those needing care, and imposing “disciplinary” measures may be functions of providers’ implicit attempts to advance “modernization” of their communities [17]. However, regardless of intent, the end result is that those who seek care but are unable to "comply" with payments are penalized, and are further marginalized by the system.

To start closing the equity gap and promote better health outcomes, facilities must stop requesting or insinuating the need to provide unnecessary items and must be funded to sufficient levels so as not to rely on payments and equipment provision by those seeking care. Where fees are still in place, costs must be transparent, waivers must be available, and those unable to pay or bring in-kind goods should never be penalized, scolded, or detained. Although larger system changes are needed to improve the working conditions of facility staff so they can provide better care, some programs that focus on provider reflection and acknowledgement of implicit bias and marginalizing practices, such as values clarification and attitude transformation exercises, have shown impact on improving respectful interactions between those needing and providing care [18, 19]. Because poor quality of care prevents care utilization, research has been primarily focused in regions of low facility-based coverage, especially for maternal and neonatal health. However, more research is needed on discrimination globally: including differential care based on class and social status [20, 21], age and perceived maturity [22], marital status [23], immigrant status [24], HIV or other infection status [25], sexual orientation [26], physical, emotional and cognitive ability [27, 28], and membership in ethnic or linguistic minority groups [29], among others.

A system which provides poor treatment for poor women strips individuals and families of their basic right to health care. The new study from Bayo et al [8] joins a small group of papers from countries with ongoing humanitarian emergencies [11, 30, 31], and demonstrates that, regardless of location, women and families demand quality and respect from the health care system. There is an urgent need to improve the *culture of care* for women and newborns – it will continue to cost lives and dignity if we do not act.

## Data Availability

De-identified data can be made available upon request by contacting the authors.

## Abbreviations

HIV: Human immunodeficiency virus
SDGs: Sustainable Development Goals
